# Aberrant DNA hypermethylation reduces the expression of the desmosome-related molecule periplakin in esophageal squamous cell carcinoma

**DOI:** 10.1002/cam4.369

**Published:** 2015-01-12

**Authors:** Takeshi Otsubo, Teruki Hagiwara, Miwa Tamura-Nakano, Takuhito Sezaki, Oki Miyake, Chihaya Hinohara, Toshio Shimizu, Kazuhiko Yamada, Taeko Dohi, Yuki I Kawamura

**Affiliations:** 1 Department of Gastroenterology, Research Center for Hepatitis and Immunology, Research Institute, National Center for Global Health and Medicine1-7-1 Kohnodai, Ichikawa, Chiba, 272-8516, Japan; 2 Communal Laboratory, Research Institute, National Center for Global Health and Medicine1-21-1 Toyama, Shinjuku-ku, Tokyo, 162-8655, Japan; 3 Department of Surgery, National Center for Global Health and Medicine1-21-1 Toyama, Shinjuku-ku, Tokyo, 162-8655, Japan

**Keywords:** Desmosome, DNA methylation, esophageal squamous cell carcinoma, periplakin

## Abstract

Periplakin (PPL), a member of the plakin family of proteins that localizes to desmosomes and intermediate filaments, is downregulated in human esophageal squamous cell carcinoma (ESCC). Little is known, however, about the molecular mechanism underlying the regulation of PPL expression and the contribution of PPL loss to the malignant property of the cancer is unclear. We demonstrated that PPL mRNA expression was significantly reduced in ESCC tissues compared with that in normal tissues. Therefore, we hypothesized that CpG hypermethylation is the cause of the downregulation of PPL. Bisulfite-pyrosequencing of 17 cases demonstrated that the frequency of *PPL* methylation was higher in ESCC tissues than in normal tissues. When human ESCC cell lines were treated with 5-aza-2′-deoxycytidine (5-aza-dC), a DNA-methyltransferase inhibitor, *PPL* transcription was induced. Human KYSE270 ESCC cells do not stratify under ordinary culture conditions and rarely produce desmosomes; however, the forced expression of PPL promoted cell stratification. PPL induction also promoted adhesion to extracellular matrix but delayed cell migration. The abundance of desmosome-like structures was greatly increased in PPL transfectant as determined by transmission electron microscopy. Very low expression of another desmosome protein EVPL in ESCC, even in PPL transfectant, also supported the significant role of PPL in desmosome formation and cell stratification. Our results first indicate that the downregulation of PPL mediated by DNA hypermethylation, which may play an important role in the loss of ESCC stratification and likely in metastatic phenotype.

## Introduction

To transfer various types of food to the stomach, the esophageal mucosa requires resistance to mechanical stress. One of the mechanisms supporting such resistance is the stratified structure of the squamous epithelial cells, which have many spines that intermingle with each other through dense desmosomes. Periplakin (PPL) is a 195-kDa, membrane-associated protein and a member of the plakin protein family, which comprises desmoplakin, envoplakin (EVPL), plectin, and bullous pemphigoid antigen 1. Plakin family proteins connect cytoskeleton elements to form intercellular junction complexes such as desmosomes 1,2. PPL is expressed in keratinized and nonkeratinized epithelial cells of the epidermis, the urinary bladder, and the oral, esophageal, and cervical mucosa 1. In epidermal epithelial cells, PPL forms stable heterodimers with EVPL that localize to the desmosomes, the interdesmosomal plasma membrane and intermediate filaments, and the plasma membrane in a manner that depends on the PPL N-terminus 3.

Despite the nearly ubiquitous presence of PPL in normal squamous cells, PPL-knockout mice do not have obvious abnormalities 4. On the other hand, proteome analysis of human esophageal cancers demonstrated that PPL was significantly downregulated in cancer tissues and was scarcely expressed in advanced-stage cancers 5. PPL expression levels are associated with cancer progression 5 and nodal metastasis 6. The loss of PPL expression was also observed in advanced-stage urinary bladder cancer 7. When PPL is expressed in early-stage cancer tissues, it localizes in the cytoplasm instead of the cell–cell boundaries 5. In pharyngeal cancer cells, PPL knockdown is related to reduced cellular movement and attachment activity 8. Although PPL downregulation is clearly associated with cancer progression and malignancy, knowledge of its contribution to the malignant property of esophageal squamous cell carcinoma (ESCC) is limited, and the molecular mechanism underlying the regulation of PPL expression is largely unknown.

CpG islands are frequently hypermethylated in cancer, leading to transcriptional repression and the subsequent reduction or loss of gene function 9. In esophageal cancer, information about cancer-associated epigenetic changes has been accumulated mainly through research on Barret esophagus and esophageal adenocarcinoma. In squamous cell carcinoma, the hypermethylation of some genes is related to clinicopathological features including poor prognosis and treatment responses 10,11. We examined the methylation status of *PPL* in ESCC and found abnormal hypermethylation in the promoter region in concordance with decreased mRNA expression levels. We restored PPL expression in an ESCC cell line and found that PPL plays a role in squamous cell stratification.

## Materials and Methods

### Patients

Paired ESCC and normal tissue specimens were obtained from 19 patients who had undergone esophagectomy or esophagogastrectomy with confirmed diagnosis of ESCC. The patients were randomly selected from those who underwent surgery from January 2013 to June 2014 at the National Center for Global Health and Medicine (NCGM). This study was approved by the research ethics committees of the NCGM (121), and informed consent was obtained from all the patients before the samples were collected. The characteristics of the samples used in each assay are summarized in Table1.

**Table 1:** Characteristics of the tumor samples included in each assay

	Total	Experiment		
		Immunohistology	RT-PCR	Pyrosequencing
Tumors with paired background mucosa	19	7	13	17
Patient gender				
Male	16	4	13	15
Female	3	3	0	2
Patient age				
Range (years)	61–85	65–85	61–85	61–85
Cancer stage				
I	7	2	5	6
II	5	2	3	5
III	6	3	5	5
IV	1	0	0	1
Tumor location				
Upper thoracic	5	0	4	4
Middle thoracic	8	4	6	7
Lower thoracic	6	3	3	6

### Cell lines and culture

Human ESCC lines KYSE70, KYSE140, KYSE150, KYSE270, KYSE410, and KYSE510 12 were obtained from the Japanese Collection of Research Bioresources Cell Bank (Osaka, Japan) on 13 August 2013. KYSE30 was obtained from HPA Culture Collections on 11 November 2010. These cell lines were maintained in Ham's F12/RPMI1640 medium containing 2% fetal calf serum. In some experiments, the cells were cultured in a 24-well plate at a density of 5 × 10^4^ cells/well and after 18 h, then treated with 5-aza-2′-deoxycytidine (5-aza-dC; Sigma-Aldrich, Inc., St. Louis, MO) for 72 h. For passage or to detach cells, 0.25% trypsin-EDTA (Life Technologies Inc, Rockville, MD) was used. Cell growth was determined by MTT assay following the vendor's protocol (Nacalai Tesque, Kyoto, Japan). For adhesion assay, cells were labeled with 3′-*O*-acetyl-2′,7′- bis(carboxyethyl)-4 or 5-carboxyfluorescein diacetoxymethyl ester (Dojindo, Kumamoto, Japan), and suspended in Dulbecco's modified Eagle's medium with 0.1% bovine serum albumin at the density of 2 × 10^5^/well of 24-well tissue culture plates coated with type I colloagen, human fibronectin or laminin (BioCoat™; Nihon BD, Tokyo, Japan). After 1 h incubation at 37°C and washing, images were captured with a fluorescent microscope. Adhered cell area was quantified using NIH ImageJ software. Cell mobility was investigated using the Cytoselect would-healing assay kit (Cell Biolabs Inc., San Diego, CA). Quantification was performed using NIH ImageJ software measuring the area covered with cells as % of whole captured square area (1.43 × 1.08 mm, designated as 100%).

### Reverse-transcription PCR

Total RNA was isolated from tissues using RNA Bee RNA Isolation Reagent (Tel-Test, Inc., Friendswood, TX). After the RNA was treated with DNase I, double-stranded cDNA was synthesized using the High-Capacity cDNA Reverse Transcription Kit (Applied Biosystems, Foster City, CA). Quantitative PCR was performed using ABI TaqMan probes (Applied Biosystems) as described previously. Threshold cycle numbers were determined using the Sequence Detector software and transformed as described by the manufacturer, with glyceraldehyde-3-phosphate dehydrogenase as the calibrator gene. The TaqMan Gene-expression assay IDs used in this study for *PPL*, and *EVPL* were Hs00160312_m1 and Hs00157430_m1, respectively.

### DNA methylation analysis

To assess the PPL methylation status, bisulfite-pyrosequencing was performed with PyroMark Gold Q24 reagents and a PyroMark Q24 pyrosequencing machine (Qiagen, Hilden, Germany). The PCR primers used in this study were 5′-AGTTGATATTGGGAGTAGGTGTTA-3′ and 5′-CAAATTCCCTAAAAACCCCTCTTAA-3′. The primer used for pyrosequencing was 5′-GGGGTTTTAGAATATAGG-3′.

### Transfection of PPL

Human HaloTag® expression vector (pFN21A HaloTag® CMV Flexi® Vector, used for mock-transfection) and human PPL HaloTag® ORF clone in pFN21A was purchased from Promega (Madison, WI) and transfected into KYSE270 cells using lipofectamine LTX reagent (Life Technologies, Inc., Rockville, MD). Stably transfected cells were isolated using a MoFlo™ XDP Cell Sorter (Beckman Coulter, Inc., Brea, CA).

### Immunohistochemical analysis

Formalin-fixed, paraffin-embedded sections of surgical specimens from patients with ESCC were deparaffinized and rehydrated. Antigen retrieval was performed using an autoclave in 10 mmol/L sodium citrate buffer. Sections were stained with hematoxylin and eosin (H&E) or anti-human PPL antibody (Sigma-Aldrich, Inc., St. Louis, MO). A diaminobenzidine staining procedure was performed using the ImmPACT™ DAB peroxidase substrate kit (Vector Laboratories, Burlingame, CA), and hematoxylin was used for counterstaining.

### Confocal microscopic analysis of cultured cells

KYSE270 cells (5 × 10^4^ cells/well) were cultured in a slide chamber (Nunc Lab-Tek; Thermo Scientific, Tokyo, Japan), washed with phosphate-buffered saline (PBS), fixed with 4% paraformaldehyde for 15 min, and permeabilized with 0.1% triton X-100 for 5 min. The cells were washed with PBS, stained with anti-human PPL antibody (Rabbit polyclonal antibody; Sigma-Aldrich) at a concentration of 3 *μ*g/mL at 4°C overnight, washed again with PBS, and incubated with Alexa Fluor 488-labeld goat anti-rabbit antibody (Life Technologies Japan, Tokyo, Japan). F-actin was visualized with Alexa Fluor 633-labeld Phalloidin (Life Technologies) at the concentration of 5 unit/mL, for 20 min at room temperature. Vectashield mounting medium containing 4′,6-diamidino-2-phenylindole (DAPI; Vector Laboratories Inc.) was used for DNA staining. Images were captured using a confocal microscopy system (FV-1000; Olympus, Tokyo, Japan). In some experiments, stained KYSE270 cells were also observed with fluorescent microscopy (IX71; Olympus).

### Transmission electron microscopy examination

Samples of human esophageal mucosa were prefixed with 2% paraformaldehyde and 2% glutaraldehyde in 30 mmol/L HEPES buffer (pH 7.4) at 4°C and then postfixed with an aldehyde-OsO4 mixture containing 1.25% glutaraldehyde, 1% paraformaldehyde, 0.32% K3[Fe(CN)6], and 1% OsO4 in 30 mmol/L HEPES buffer (pH 7.4) for 1 h at room temperature. The fixed blocks were dehydrated and then embedded in Quetol 812 (Nisshin EM, Tokyo, Japan). The blocks were sectioned to a 70-nm thickness with an ultramicrotome (Leica EM UC7; Leica, Wien, Austria). The ultrathin sections were contrasted with EM stainer (Nisshin EM) and examined with an electron microscope (JEM-1400; JEOL, Tokyo, Japan). Cells on culture dishes (Nunclon™Δ Surface; Nunc, Roskilde, Denmark) were washed briefly with PBS and fixed with aldehyde-OsO4 mixture [1.25% glutaraldehyde, 1% paraformaldehyde, 0.32% K3[Fe(CN)6], and 1% OsO4 in 30 mmol/L HEPES buffer (pH 7.4)] for 1 h at room temperature as previously reported method 13 with slight modification. Fixed cells were washed with Milli Q water for three times and stained en bloc with EM stainer diluted to 1/4 in 50% EtOH for 1 h, dehydrated in an ethanol series, and then embedded in Quetol 812. The blocks were trimmed to an area of ∽0.5 mm and serially sectioned at 70 nm thickness with an ultramicrotome (Leica EM UC7; Leica). The thin sections were examined with an electron microscope (JEM-1400; JEOL).

### Statistical Analysis

The data were expressed as the mean ± standard deviation, and the results were compared by paired or unpaired Student's *t* test using the Prism 6 statistical program (GraphPad Software, Inc., La Jolla, CA). All tests were two-tailed, and *P* < 0.05 were considered significant. Correlations were tested with a two-tailed Pearson calculation.

## Results

### PPL expression was lower in ESCC than in normal esophageal mucosa

**Figure 1:**
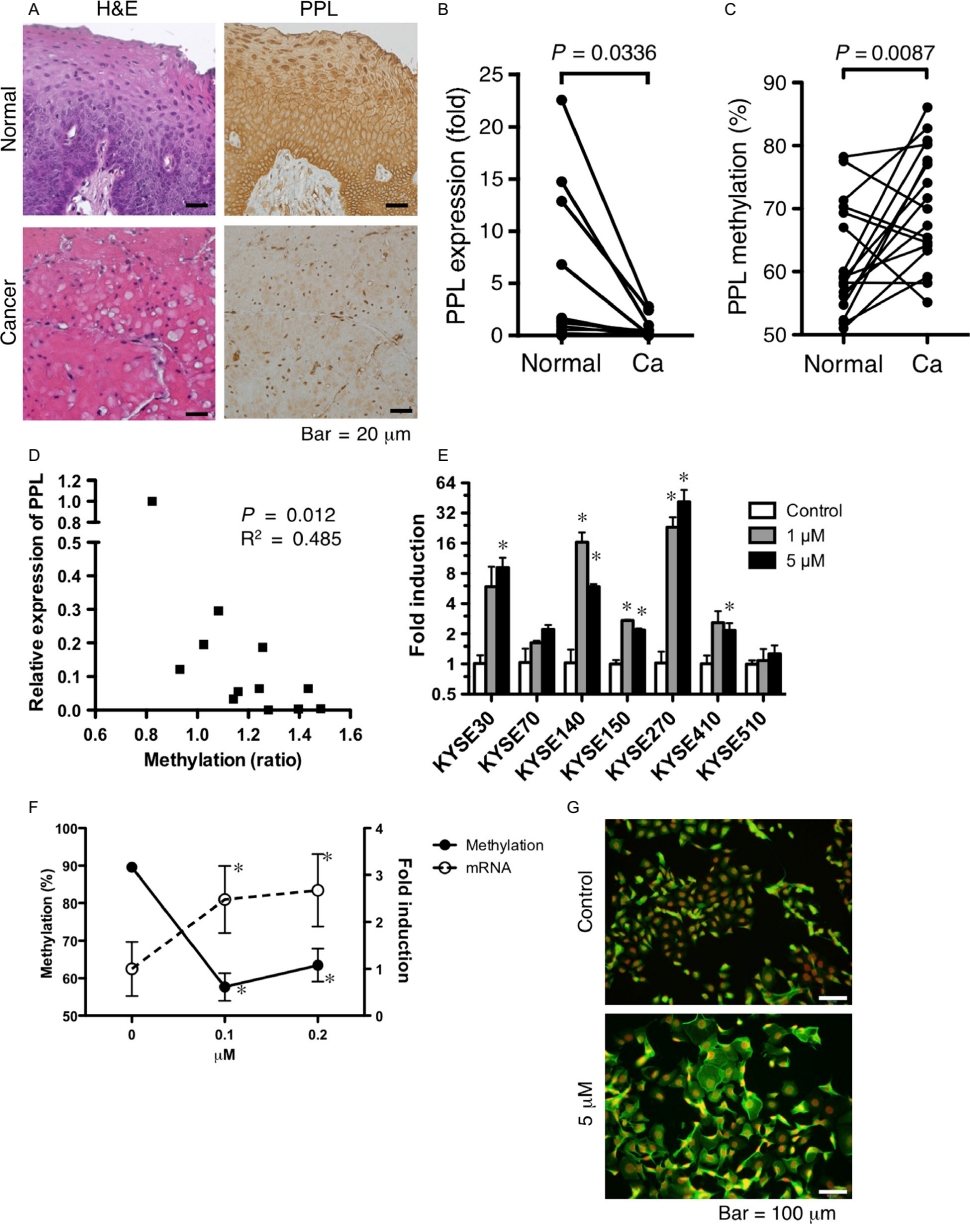
PPL expression was silenced by DNA methylation in ESCC. (A) Typical images of formalin-fixed, paraffin-embedded samples of ESCC, and adjacent noncancerous mucosa (normal) stained with H&E or anti-PPL antibody. (B) PPL transcript levels in paired samples from 13 ESCC samples were determined by RT-PCR. Data indicate expression relative to the mean levels of normal tissues. (C) DNA methylation of *PPL* as determined by pyrosequencing of paired samples from 17 patients with ESCC. (D) Expression of PPL in ESCC relative to that in normal tissue plotted against the change in DNA methylation (ratio of tumor to normal tissue) in each paired sample. (E) PPL mRNA induction in ESCC cell lines after treatment with 1 or 5 *μ*mol/L of 5-aza-dC. The fold increase in induction in the treated cells relative to that in the untreated cells is shown for each cell line. Data are shown as mean + SD of duplicated assays. *Difference from untreated cells was statistically significant (*P* < 0.05). (F) KYSE270 cells were treated with the indicated concentrations of 5-aza-dC. The levels of methylation of PPL DNA (left *Y* axis, solid lines) and mRNA (right *Y* axis, dotted lines) are shown. Data are shown as mean ± SD of triplicated assays. *Difference from untreated cells was statistically significant (*P* < 0.05). (G) KYSE270 cells were treated with 5 *μ*mol/L of 5-aza-dC, and stained with anti-PPL antibody (green) and DAPI for nuclear staining (red). ESCC, esophageal squamous cell carcinoma; PPL, periplakin.

First, we confirmed the previous reports 5,6 that PPL expression in ESCC was reduced compared with that in normal esophageal mucosa. The immunohistochemical results showed that all the normal esophageal squamous cells expressed PPL (Fig.1A). Three of seven ESCC samples had significantly reduced (less than 80% of cells were PPL positive, histological score = 1) PPL expression, and other four had scarce (less than 10% of cells were PPL positive, histological score = 0) PPL expression (Fig.1A). When compared with normal tissue (100% of cells were PPL positive, histological score = 2 in all cases), downregulation of PPL (average ± SD was 0.428 ± 0.53) in tumors was significant (*P* = 0.0009). In accordance with those results, the PPL mRNA levels determined by RT-PCR were significantly decreased in the cancer tissues compared with those in the normal mucosa (Fig.1B).

### DNA hypermethylation mediated transcriptional suppression of *PPL* in ESCC

Promoter methylation is a frequent event in cancers including ESCC and is associated with transcriptional repression and the subsequent reduction or loss of gene function. We examined whether methylation of the *PPL* promoter could explain the reduced PPL expression in the ESCCs. We determined the DNA methylation levels of CpG islands in the PPL promoter using pyrosequencing and compared them between the ESCC and paired normal tissue samples from 17 patients. The average methylation level was 61.8 ± 8.7% and 70.5 ± 9.3% (mean ± SD) in the normal and tumor samples, respectively. A paired *t*-test indicated that the tumor DNA was significantly more methylated than that in the normal tissues (Fig.1C). In addition, the relative PPL expression levels in tumors were negatively correlated with the increase in DNA methylation levels (Fig.1D), indicating that aberrant hypermethylation of the *PPL* promoter in ESCC was likely the cause of the downregulated expression.

DNA hypermethylation also silenced *PPL* expression in the ESCC cell lines. We determined endogenous PPL mRNA expression levels in seven ESCC cell lines and compared them with the levels in cells treated with 5-aza-dC. In all the cell lines, treatment with 5-aza-dC resulted in the trend of increased the expression of PPL mRNA and reached statistically significant levels in five cell lines (Fig.1E). The DNA methylation levels of the untreated KYSE270 ESCC cells were as high as 90%, and those of the cells treated with low concentration of 5-aza-dC (0.1 *μ*mol/L) decreased to around 60%. Higher concentration of 5-aza-dC did not further decrease methylation levels. Similar to that in the ESCC tissue samples, the decreased DNA methylation was associated with the induction of PPL transcripts (Fig.1F) and protein expression (Fig.1G). The methylation and expression levels strongly suggest that PPL expression was silenced by DNA hypermethylation in the PPL promoter in both the tumor samples and the tumor cell lines.

### Forced PPL expression induced epithelial cell stratification and the piling up of cancer cells

**Figure 2:**
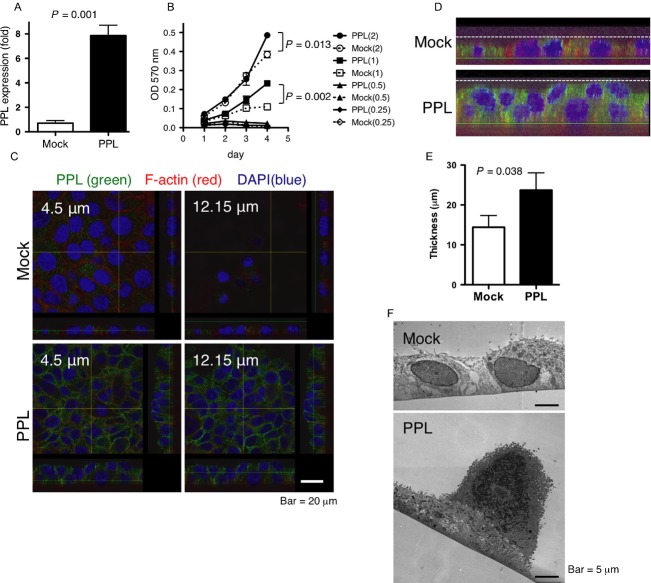
Forced PPL expression promoted cell stratification (A) PPL mRNA levels in mock-transfected and PPL-transfected KYSE270 cells. Data are shown as mean + SD of triplicate assays. (B) Growth curves of PPL-transfected and mock-transfected KYSE270 cells as determined by MTT assay. Culture was started at cell density of 0.25, 0.5, 1, or 2 × 10^3^/well. Data are shown as mean ± SD of triplicate assays. (C) Cells were cultured for 2 days, and PPL (green) and F-actin (red) were visualized with nuclear staining (blue). Images were obtained at the indicated *Z*-axis level from the basal level (=0) of the cells. (D) Magnification of the *Z*-stack image at the bottom of panel C with modified brightness and contrast to show shapes of cells. The dotted line and the green line indicate the maximum thickness and basal level of the cells, respectively. (E) The maximum thickness of the cultured-cell sheet as determined in D. Data are shown as mean + SD of three images similar to the one shown in (D) obtained from different cultures. (F) A typical TEM image of mock-transfected and stratified colonies of PPL-transfected cells. PPL, periplakin.

To further understand the significance of PPL defects in ESCC, we investigated the effect of forced PPL expression in the ESCC cell line KYSE270. We prepared a PPL-transfectant cell line and a mock-transfectant cell line. The PPL expression level in the PPL transfectants was around eightfold higher than that in the mock transfectants (Fig.2A). Initially, we tested whether PPL expression in the KYSE270 cells affects cell viability or growth. When the cultures were started with a low cell density (0.25–0.5 × 10^3^ cells/well), the growth of the PPL transfectants was almost the same as that of the mock transfectants on day 3, after which neither cell type increased its density further (Fig.2B). When the cultures were started at higher cell densities, the PPL and mock transfectants proliferated at a similar rate for 3 days; after 4 days, however, the PPL transfectants increased their density more than the mock transfectants (Fig.2B).

The appearances of the PPL-transfectant and mock-transfectant cultures were different. The original KYSE270 cells and the mock transfectants grew mostly in a spreading shape on the culture dishes, forming a monolayer sheet with no stratification (Fig.2C). In contrast, the PPL transfectants often piled up, forming stratified layers of ∽2–3 cells, and they reached a higher density in culture compared with the mock transfectants (Fig.2C). The piled cells could be seen in the confocal microscope images at different levels on the *Z* axis (Fig.2C), which showed the PPL transfectants piled up into multiple layers and the mock transfectants remaining in a monolayer. Moreover, the images of the *Z* stacks of PPL-expressing cells clearly demonstrated a stratified structure (Fig.2D). We used the images to measure the maximum thickness of the cells on the culture dish. The thickness of the PPL transfectants was significantly greater than that of the mock transfectants (Fig.2E). We further confirmed the piling of the cells in the PPL-transfectant cultures by transmission electron microscopy (TEM; Fig.2F), which detected stratified cells in the PPL-transfected cultures but not in the mock-transfected cultures.

**Figure 3:**
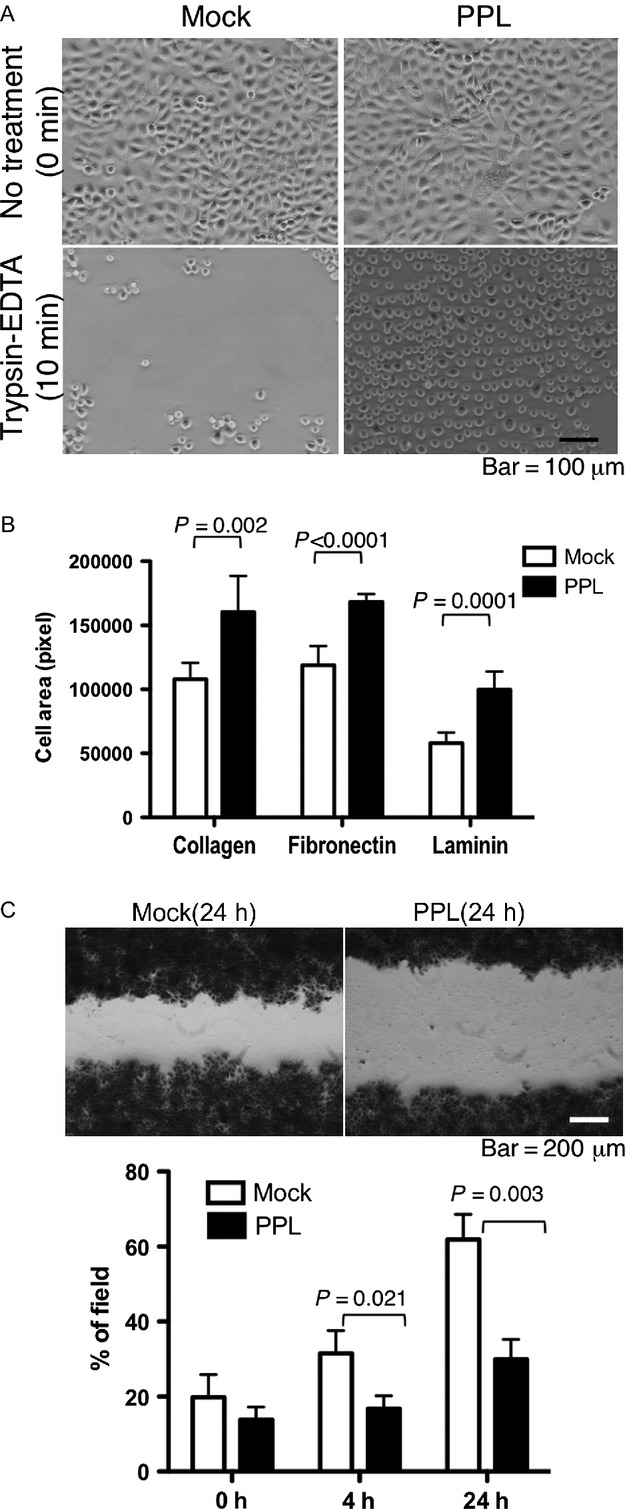
Forced PPL expression promoted cell adhesion. (A) Cell detachment assay. The same numbers of mock-transfected or PPL-transfected KYSE270 cells were cultured in 10-cm dishes for 24 h and then treated with 1 mL trypsin-EDTA for 10 min no treatment, 0 min. (B) Cell adhesion assay. Mock-transfected or PPL-transfected KYSE270 cells were fluorescent-labeled, and incubated for 1 h in plates coated with the indicated extracellular matrix. The area of adherent cells was measured. Two random fields were counted in three separate wells, and the results were shown as mean + SD. (C) Migration assay. Mock-transfected or PPL-transfected KYSE270 cells were grown to confluence in a 24-well plate with an insertion to keep a cell-free area. After the removal of the insertion, cells were cultured for indicated times and images were captured. Cell migration was quantified by measuring the % of area covered with cells. Data are shown as mean + SD of triplicate assays. PPL, periplakin.

The PPL transfectants adhered more to the culture plates than the mock transfectants did. When sparsely cultured cells were treated with trypsin and EDTA, most of the mock transfectants became detached within 10 min, whereas the PPL transfectants remained attached (Fig.3A). We tested adherence to various extracellular matrices and found that the PPL transfectants adhered more strongly to collagen, fibronectin, or laminin-coated plate compared with the mock transfectants (Fig.3B). In addition, the wound-healing assay showed that wound closure of the PPL-transfected KYSE270 cells was remarkably delayed compared with that of the mock-transfected cells (Fig.3C). The enhanced stratification and adherence, and attenuated migration of the PPL transfectants compared with those of the cells not expressing PPL at high levels indicate that PPL plays significant roles in cell adhesion and mobility.

### PPL transfection induced the formation of desmosome-like structures

**Figure 4:**
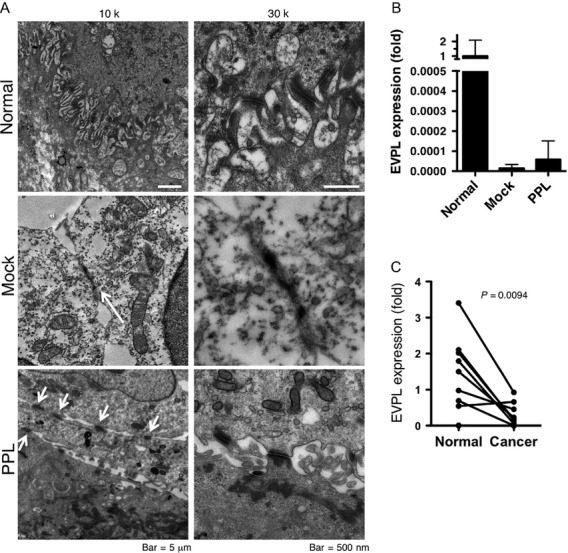
Forced PPL expression induced desmosome-like structures. (A) TEM images were obtained from normal human esophageal mucosa (normal) and mock-transfected or PPL-transfected KTSE270 cells at 10,000× or 30,000× magnification. Desmosomes were frequently found in normal tissues and PPL-transfected cells (arrows). In the images of the mock-transfected cells taken at 10,000× magnification, the arrow indicates adhesion plaque-like structures, which was not identified as a desmosome at higher magnification. (B) Expression of envoplakin (EVPL) in mock- or PPL- transfected KYSE270 cells. EVPL mRNA levels were shown as fold expression of the levels to normal esophageal mucosa (average of 13 mucosa = 1). Data are shown as mean + SD of three assays. (C) Expression of envoplakin (EVPL) in ESCC tissues with paired normal mucosa. PPL, periplakin; ESCC, esophageal squamous cell carcinoma.

PPL is one of the proteins that make up desmosomes, so we looked at the cell–cell adhesions in PPL transfectant further in TEM images (Fig.4A). In the normal esophageal mucosa, the squamous cells had many desmosomes at intercellular junction complexes between intermingled spines. The KYSE270 cells did not stratify under our culture conditions, and rarely contained desmosomes. Although there were a few attachment-like structures with high electron density, the detailed morphology of the KYSE270 cells was different from that of the normal esophageal squamous cells. In contrast, the forced expression of PPL dramatically increased the abundance of desmosome-like structures, which is apparently similar to those in normal cells. This observation suggested that the downregulation of PPL mediated by DNA hypermethylation might play an important role in the loss desmosome in ESCC. EVPL, another desmosomal protein, forms heterodimer with PPL in epidermal squamous cells, and its somatic mutation is linked to ESCC 14. To examine the contribution of PPL and EVPL to the formation of desmosomes, the transcript levels of EVPL were measured in the mock-transfected or PPL-transfected KYSE270 cells and compared with those in normal esophageal mucosa. The expression of EVPL was strikingly downregulated in both mock and PPL transfectants, and there was no difference between mock and PPL transfectants, indicating that EVPL expression was not induced in PPL transfection. Therefore, even when EVPL is almost absent, the PPL induction is able to recover the desmosomes. The decrease in EVPL in tumor tissues was also generally seen in ESCC cases (Fig.4C).

## Discussion

ESCC is an aggressive form of cancer and one of the most frequent types of cancer in the world and especially in Asian countries 15. Environmental factors such as smoking and alcohol consumption are the risk factors for ESCC in Western countries 16, and the consumption of hot beverages has been shown to be a major risk factor in the East 17. The environmental risk factors affect the condition of the epithelial mucosa, inducing mutations and epigenetic changes, which accumulate, induce mucosal dysplasia, and eventually develop into invasive squamous cell carcinoma. During that process, gene silencing via DNA hypermethylation of CpG islands in gene promoters often takes place. We found that PPL, a desmosome protein, was one of the targets of aberrant DNA hypermethylation in ESCC. A previous global study using bead arrays to detect promoter-DNA methylation demonstrated that a total of 37 CpG sites were differentially methylated between esophageal squamous cell tumors and background mucosa 11. PPL was not specifically listed, however, in that report or other previous reports. One reason may be the high methylation status of the PPL promoter region in the normal/background mucosa; the methylation levels were more than 50% in all the normal tissue samples in our study. Although the increase in the methylation levels, which reached 70%, in the tumors was not very dramatic, the difference in the mRNA levels between the tumor and control tissues was very clear. The phenomenon was recapitulated in our experiment with the ESCC cell lines. The methylation levels were about 90% in the KYSE270 cells, and the 5-aza-dC treatment reduced them to around 60%. Increasing the concentration of 5-aza-dC did not decrease the methylation levels further. On the other hand, a clear recovery of PPL mRNA was observed in association with the decreased DNA methylation, suggesting that the PPL promoter is likely to be around 50% methylated even in normal cells and that additional hypermethylation in tumors affects PPL expression. Such epigenetic alteration might have been overlooked by global analyses, depending on the conditions used to identify hypermethylated genes. We found that the recovery of PPL via gene transfection induced the formation of desmosome structures and cell attachment in ESCC cells. Because the levels of PPL mRNA increased in the PPL-transfected cells by up to eightfold, which is comparable to the difference between cancer and background mucosa, we think that similar changes in biological function based on changes in PPL expression between normal mucosa and tumors can be expected. As a matter of fact, the presence of aberrant DNA hypermethylation in noncancerous esophageal mucosa of ESCC cases was reported in association with smoking history 18. As shown in case of PPL in our study, modest but significant additional DNA methylation to risky background esophageal mucosa with hypermethylation may trigger the development of ESCC.

Desmosomes consist of many proteins, and a previous study reported that a lack of *PPL* alone did not affect desmosome formation in skin epithelial cells 4, although the condition of the esophageal mucosa was not clearly described. Therefore, we had not expected the forced expression of *PPL* alone to induce such a dramatic change in the formation of desmosome-like structures and cell adhesion. In the squamous cells of the skin or urinary tract, PPL colocalizes with EVPL, another plakin family protein. A previous report found that in the esophagus, the EVPL level was highest in the outermost layers, whereas PPL is expressed in all the suprabasal layers 1. In our study, the expression of EVPL was significantly downregulated in tumors compared with that in the normal mucosa. We have not investigated the mechanism for down regulation of EVPL in this study; however, absence of promoter CpG island in EVPL gene suggests that its downregulation may not be due to DNA hypermethylation. Those results suggest that in esophageal squamous cells, the presence of PPL is sufficient for desmosome formation even in the absence of EVPL. The strong roles of PPL in desmosome formation and cell stratification may therefore be a characteristic of esophageal squamous cells. When the loss of PPL in the cancer cell lines was compensated for, by gene transfection, the cells became smaller and acquired a more cylinder-like shape with the ability to grow in stratified layers, indicating that cells gained the ability to differentiate to squamous epithelial cells. The preference of the PPL transfectants for adhesion to extracellular matrix also supports the propensity of epithelial differentiation. We think that the higher growth rate of the PPL transfectants compared with that of the mock transfectants may be a result of the acquisition of the ability to form columnar epithelium with stratification, which enabled the PPL transfectants to grow to a higher density in the culture dish in the later stages of the growth curve, when the cells were at higher density. Similar results were previously reported in experiments that eliminated PPL by using siRNA in pharyngeal squamous cancer cell lines 8. The authors found that PPL knockdown decreased tumor cell growth, adhesion to ECM. Although the promotion of cell growth in the presence of PPL may appear to conflict with the gain of other features of differentiation, it does not necessarily indicate that the PPL transfectants are able to proliferate in vivo more successfully than the original ESCC cells. Furthermore, distinct from the previous report using pharyngeal cancer cells using siRNA of PPL 8, we found that PPL induction inhibited migration of ESCC, which is a feature of epithelial–mesenchymal transition, the hallmark of metastatic cancer. Loss of function of adherence junction due to downregulation of another desmosomal component desmocollin 2 promoted cell aggressiveness and migration of ESCC 19, and its reduction is also associated with tumor progression and poor prognosis of ESCC 20. In this sense, PPL loss induced by promoter hypermethylation may contribute to the malignancy of metastatic ESCC.
